# Systematic review and meta-analysis of MRI-based sex differences in the human fetal brain

**DOI:** 10.1162/IMAG.a.1295

**Published:** 2026-07-06

**Authors:** Max A. Paulus, Jacob E. Schuman, Lise Eliot

**Affiliations:** Chicago Medical School, Rosalind Franklin University of Medicine & Science, North Chicago, IL, United States; Stanson Toshok Center for Brain Function and Repair, Rosalind Franklin University of Medicine & Science, North Chicago, IL, United States

**Keywords:** brain development, cerebellum, functional connectivity, gender, in utero, placenta

## Abstract

Sex differences in child neurobehavioral health suggest that male and female brains differ early in development. We took advantage of recent advances for in-utero magnetic resonance imaging (MRI) to conduct a pre-registered systematic review and meta-analysis of sex differences in brain structure and network connectivity of human fetuses. PubMed literature searching yielded 4,738 studies published between 2002 and 2025. All studies were screened by two independent reviewers and included if either structural or functional MRI was used to image brains of healthy human fetuses in utero and any results were reported stratified by sex. After title and abstract screening, 545 studies remained for full-text screening, resulting in 34 total studies meeting inclusion criteria. Analysis focused on 28 of these that reported sex-disaggregated data on the same measure across three or more independent samples. Pooled effect sizes revealed significantly larger male brains based on both linear measures (cerebral fronto-occipital and biparietal diameters and corpus callosum length) and global volumes (intracranial, total brain, lateral ventricles) by the start of the third trimester. Among 11 studies reporting brain growth trajectories, a majority reported faster growth in males. Among nine studies measuring functional connectivity using resting state functional MRI (rs-fMRI), six reported no significant sex differences and the others reported sporadic differences that were not replications. Together with large ultrasound studies, this review demonstrates larger brain size and faster brain growth in human males compared to females beginning in the second trimester, comparable to overall body size and other internal organ volumes. However, existing MRI and ultrasound research has not identified specific brain regions that differ disproportionately between male and female fetuses or any reliable sex differences in functional connectivity. Faster fetal growth in males, including the brain, does not readily explain neonatal male vulnerability and appears to be a product of genetic, rather than hormonal influences. These findings provide a reference for the emergence of brain sex differences later in development.

## 1 Introduction

Sexual differentiation begins in the first trimester of human gestation. Between 41- and 44-days post-conception, competing cascades of gene expression are launched in the primordial gonad, favoring testes formation in the presence of SRY gene expression and ovary formation in its absence. When testes form, they begin secreting anti-Müllerian hormone and testosterone, which are largely responsible for differentiation of the male urogenital system. When ovaries form, the absence of these hormones leads to differentiation of a female-typical urogenital system (Ray & Racine, 2025).

Compared to the genitalia, sexual differentiation has been little studied in other organs, with the notable exception of the brain. Fueled by interest in adult brain sex differences, many researchers have turned their attention to sex differences in fetal brain development, hypothesized to arise from variations in prenatal testosterone exposure (Bakker, 2022; Knickmeyer & Baron-Cohen, 2006). Other rationale for studying fetal brain sex differences derives from well-described differences between male and female infants in mortality and morbidity (Di Renzo et al., 2007; Vatten & Skjaerven, 2004; Weng et al., 2015), especially involving neurobehavioral deficits (DiPietro & Voegtline, 2017). Relative to females, males are more vulnerable to fetal demise, preterm birth, low birth weight, and intraventricular hemorrhage. When born preterm, boys are less likely to survive than girls, and have poorer neurodevelopmental outcomes, including higher rates of cerebral palsy (Romeo et al., 2023). Overall, boys are more likely than girls to be diagnosed with autism (Loomes et al., 2017), intellectual disability, and ADHD (Zablotsky et al., 2019) in early life. All of these disparities suggest that cerebral maturation may proceed more slowly in males, as proposed more than 50 years ago by Taylor (1969), an influential hypothesis that has driven considerable research on early human development.

With regard to fetal behavior, modest evidence also supports the notion of faster CNS maturation in females. Although there is no sex difference in fetal movements or auditory perception (DiPietro et al., 2010; Hepper et al., 2012), female fetuses between 30 and 35 weeks of gestation demonstrate faster habituation to vibroacoustic stimulation, a finding that has been replicated across three different cohorts (Hepper et al., 2012; McCorry & Hepper, 2007). Habituation is a simple but important form of learning thought to depend on higher brain centers, so this difference suggests that cortical development may proceed more rapidly in female fetuses. One small study of spontaneous EEG in full-term neonates supports earlier brain maturation in females (Thordstein et al., 2006). Studies of preterm infants using amplitude-integrated EEG signals similarly suggest a maturational advantage for females born between 28- and 31-weeks gestational age, but the differences were mostly non-significant (Griesmaier et al., 2014), especially among medically-healthy preterm infants (Olischar et al., 2013).

Evidence for behavioral sex differences remains modest in newborns, but is stronger later in the first year of life. Decades of study have revealed no reliable differences in neonatal motor (de Vries et al., 1988; Fischel, 1982; Korner et al., 1981; Partington et al., 1971) or social behavior (Karson et al., 2025), although girls do appear to orient more strongly to both animate and inanimate stimuli at birth (Lundqvist & Sabel, 2000). However, by 12 months post-natal, small sex differences have been documented in various behaviors, including activity level (Campbell & Eaton, 1999), face processing (McClure, 2000), toy preference (Todd et al., 2018), vocabulary (Wallentin & Trecca, 2023), and other cognitive (Alexander & Wilcox, 2012) skills. Given the high cultural salience of gender and intensive social interactions during infants’ first year, it is difficult to know the degree to which these behavioral sex differences are a result of innate brain differences versus learned/neuroplastic processes (Johnson et al., 2014; Karson et al., 2025; Mascaro et al., 2017).

Analysis of fetal brain sex difference may help answer such questions, as well as lead to a better understanding of male vulnerability in the preterm and neonatal periods. Until recently, fetal brain research was limited to postmortem or live ultrasound measures, but with advances in prenatal MRI techniques, particularly involving motion correction and faster image acquisition, research over the past 15 years has allowed more precise study of fetal brain tissues (Studholme, 2011). Such advances have led to a recent surge of research on fetal brain development, some of which has included reporting on sex differences in brain structure, function, and growth.

In this study, we systematically reviewed the extant literature for MRI studies that have assessed sex differences in healthy human fetal brain structure, function, and growth. Based on prenatal ultrasound (Broere-Brown et al., 2016; Galjaard et al., 2019; Melamed et al., 2013) and neonatal MRI studies (Dean et al., 2018a; Gilmore et al., 2007; Khan et al., 2024; Knickmeyer et al., 2014), we hypothesized that male fetal brains would be larger than female brains, and sought to determine when this divergence emerges. Our search terms also included key brain structures, to test whether specific brain areas diverge disproportionately in size or activity between male and female fetuses. We captured both structural and functional MRI studies, as both methods have been added to the arsenal of prenatal brain investigations and can help assess whether neural circuitry differs meaningfully between male and female infants before birth.

## 2 Methods

This study was pre-registered in the PROSPERO database of prospectively registered systematic reviews (Schuman et al., 2024).

### 2.1 Search strategies and criteria

To capture all published studies that reported MRI brain measures separately in male and female fetuses, a literature search was conducted in PubMed for English language studies indexed through May 4, 2025 using the following search terms: (Brain*[tiab] OR Cerebr*[tiab] OR Corpus Callosum[tiab] OR Connectivity[tiab] OR Connectome[tiab] OR Superior Longitudinal Fasciculus[tiab] OR Corticospinal[tiab] OR Hemispher*[tiab] OR Internal Capsule[tiab] OR White Matter[tiab] OR Hippocamp*[tiab] OR Lateraliz*[tiab] NOT Palsy[tiab]) AND (Neuroimaging[tw] OR MRI[tw] OR Magnetic Resonance[tw] OR DTI[tw] OR diffusion tensor[tw] OR Fractional Anisotropy[tw] NOT Echocardio*[tiab] NOT Doppler[tiab] NOT Ultraso*[ti]) AND (Sex OR Gender OR Male OR Female OR Boy OR Girl OR Dimorph* NOT “Gender Identity” NOT “Sexuality”) AND (Fetal OR Fetus OR Prenat* OR Gestat*) NOT (Autop*[tiab] OR Hemorr*[ti] OR Cardi*[ti] OR Heart[ti] OR Arter*[ti] OR Lesion*[ti] OR Tumor*[ti] OR Treat*[ti] OR Sheep[tiab] OR Lamb[tiab] OR Mouse[tiab] OR Mice[tiab] OR Murine[tiab] OR Rat[tiab] OR Rodent[tiab] OR Pig[tiab] OR Piglet[tiab] OR Porcine[tiab] OR Animal[ti]).

**Fig. 1.**
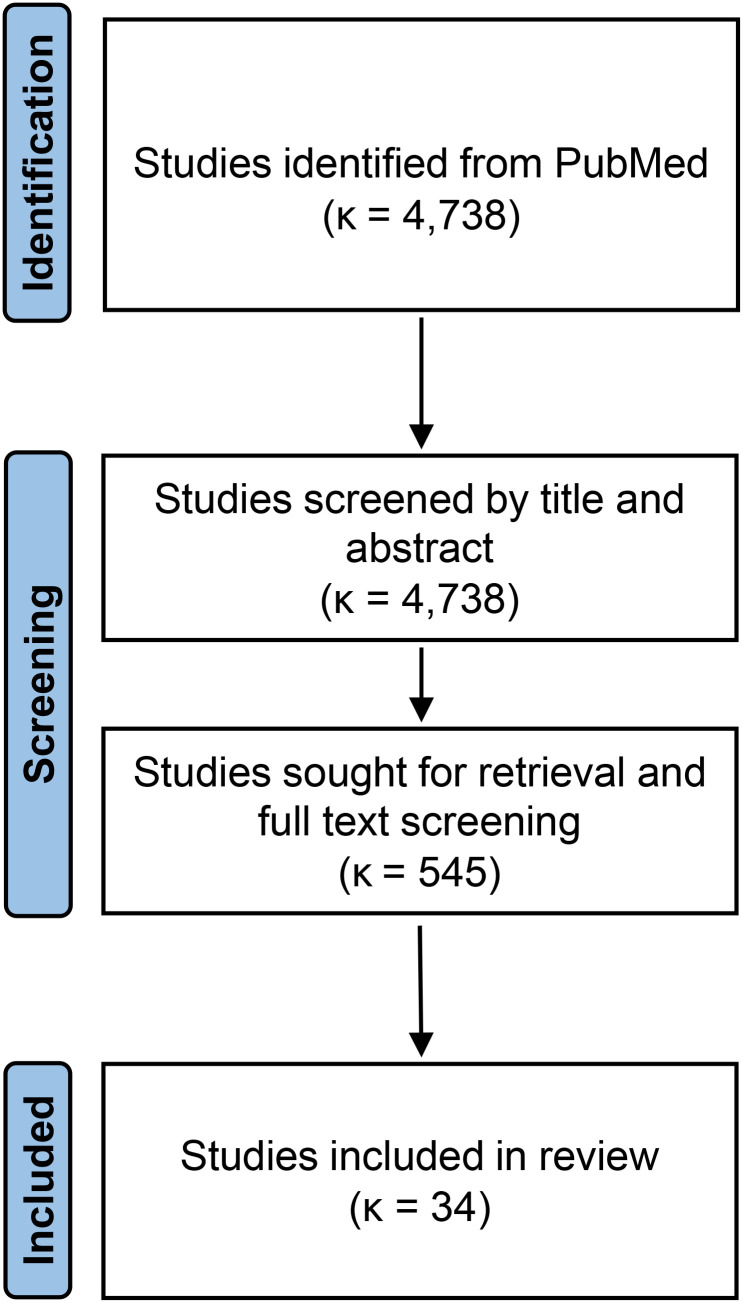
Summary of the steps in systematic literature search and screening. Every study was screened by at least two independent reviewers. See Section 2.1 for search terms and inclusion and exclusion criteria.

As shown in Figure 1, this search strategy yielded 4,738 non-duplicated studies published from 2002 through 2025. Titles and abstracts were then screened per the Preferred Reporting Items for Systematic Reviews and Meta-Analyses (PRISMA) guidelines (Moher et al., 2009) by two independent reviewers using Rayyan software (Ouzzani et al., 2016). Studies were included if either structural or functional MRI was used to image brains of healthy human fetuses in utero and at least some results were reported stratified by sex. Post-mortem and case reports were excluded, along with MRI measures outside of structure or neural function (e.g., relaxometry, or reported correlations between MRI measures and infant behavior or maternal measures) and any data from fetuses diagnosed with congenital malformations or developmental disorders. We also excluded two studies (Zhang et al., 2011; Kyriakopoulou et al., 2014) whose samples were completely subsumed in subsequent larger studies (Meng et al., 2012; and Kyriakopoulou et al., 2017, respectively). Any discrepancies between the two independent reviewers were resolved after unblinding. After title and abstract screening, 545 studies remained for full-text screening. Finally, the literature search was complemented by unsystematic searches and reference harvesting from included studies and relevant review articles.

### 2.2 Data analysis

For studies providing full quantitative data, sex difference pooled effect sizes (Hedges’s g) were computed based on the mean and standard deviations for separate male and female groups or the t- or F-values for such comparisons, with a positive value indicating higher score in males. Hedges’s g was used in lieu of Cohen’s d due to the small sample sizes typical of fetal studies (Borenstein et al., 2009). For studies in which effect sizes were stated to be “non-significant” and could not be calculated from the published data, we followed the conservative approach of assigning Hedges’s g = 0 (Rosenthal, 1991). To ensure this imputation did not unduly distort the results, a sensitivity analysis was performed by running the meta-analyses with and without these “qualitative” studies, as indicated.

When multiple studies with overlapping samples were reported by the same research team, we analyzed only the later results, unless the earlier study reported data that were explicitly not included in the later one (e.g., Andescavage et al., 2017a, 2017b; Lu et al., 2021, 2022).

For any measure reported across three or more studies, meta-analysis was conducted using a random-effects model and Comprehensive Meta-Analysis (CMA) version 4.0 software. Statistical significance was evaluated using a z-test, with two-tailed significance set at p < 0.05. For studies that scanned any of the fetuses at more than one gestational age, sample size refers to the number of individual fetuses, not the total number of scans in each group.

## 3 Results

**Fig. 2.**
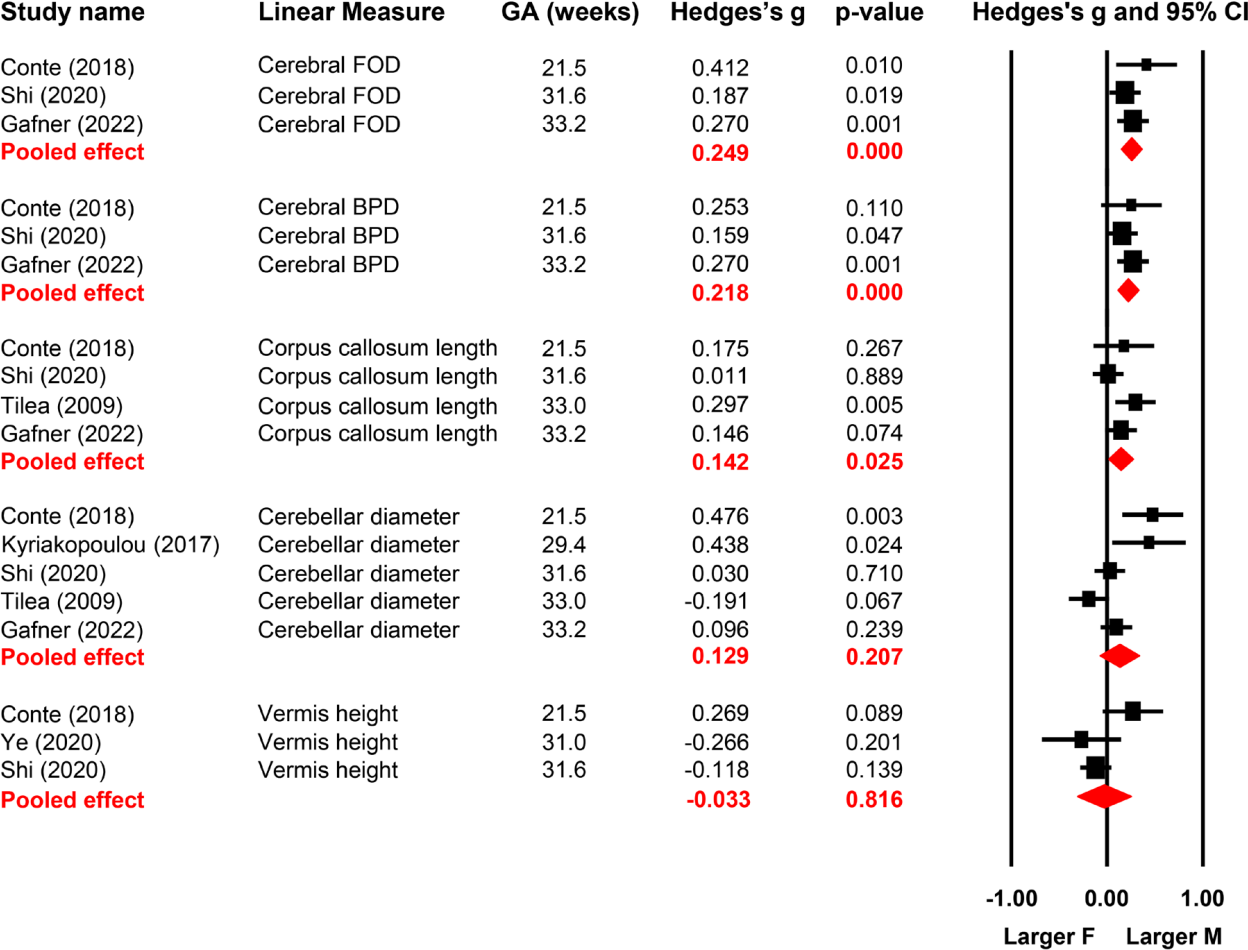
Meta-analyses of fetal brain sex differences based on linear MRI measures. Sex difference effect sizes (Hedges’s g values) and 95% confidence intervals (CI) for linear brain measures ordered by pooled effect size (red), with studies ordered by mean or median gestational age (GA). The width of red diamonds corresponds to the confidence interval for the pooled effects. All measures were significantly larger in male fetuses except cerebellar diameter and vermis height. Abbreviations: FOD = fronto-occipital diameter; BPD = biparietal diameter; F = female; M = male.

**Fig. 3:**
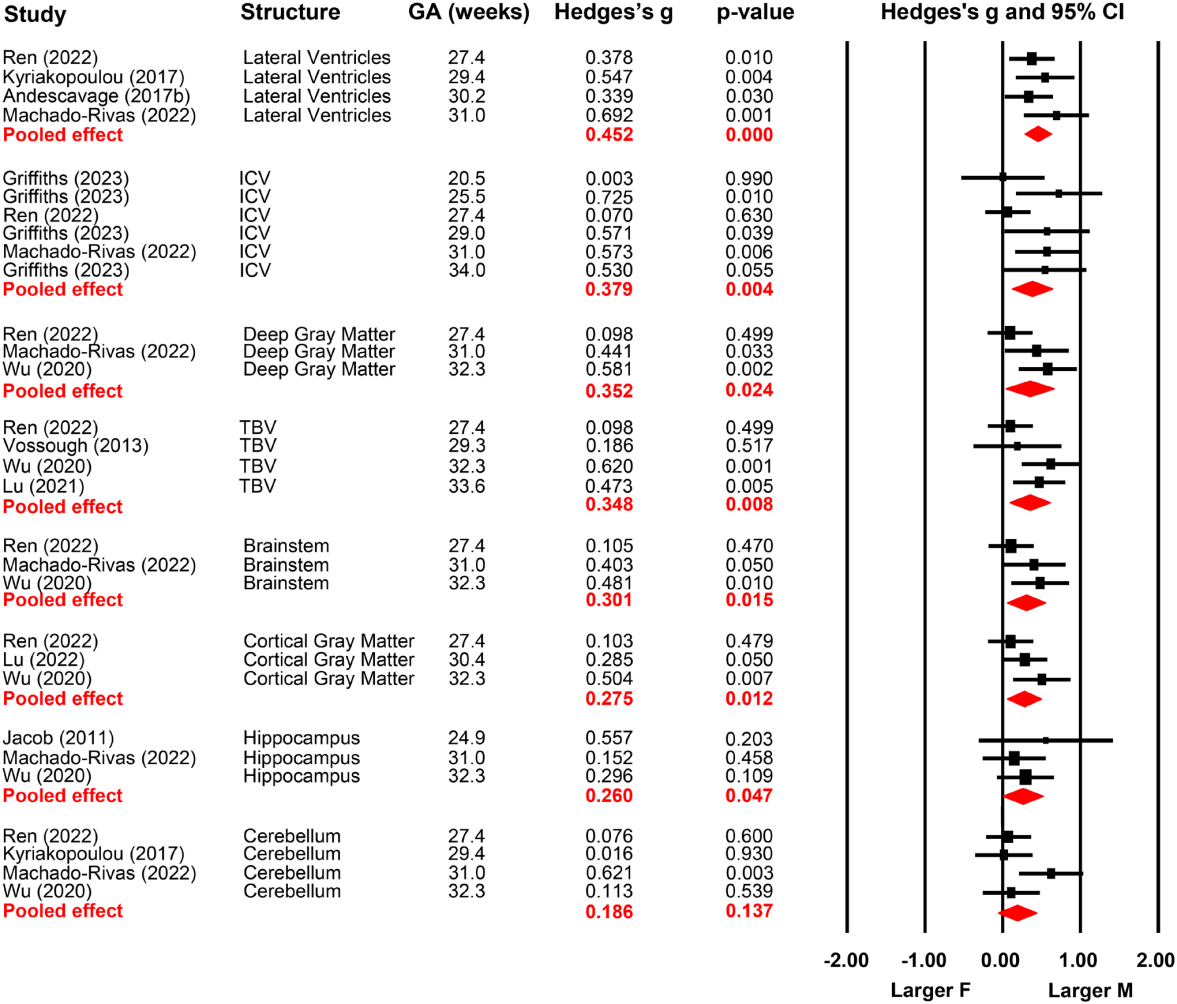
Meta-analysis of MRI-based fetal brain volume sex differences. Sex difference effect sizes (Hedges’s g values) and 95% CI for structural volumes ordered by pooled effect size (red), with studies ordered by mean or median gestational age GA. The width of red diamonds corresponds to the confidence interval for the pooled effects. All volumes were significantly larger in male fetuses except for the cerebellum. Abbreviations: ICV = intracranial volume; TBV = total brain volume; F = female; M = male.

Out of 4,738 total articles screened, 34 studies met inclusion criteria (Table 1). Of these, our analysis focused on the 28 studies that reported sex-disaggregated data on the same MRI measure across three or more studies or independent samples. Comparisons of male versus female fetuses were partitioned into four clusters according to dependent measure: linear brain measures (Fig. 2 and Table 2A), volumetric measures (Fig. 3 and Table 2B), growth measures (Table 3), or rs-fMRI connectivity (Table 4). Mean or median fetal age ranged across studies from 17 to 33 weeks, with most studies centered on fetuses of approximately 30 weeks’ gestational age.

**Table 1.** Studies reporting on sex difference in fetal brain MRI measures.

Study	#M, F	GA	Measures reported by sex	Sex similarities & differences
Andescavage (2017a)	87, 79	18 - 39	Volumes and growth of cerebral cortex, cerebellum, CGM, DGM, WM	No significant sex differences.
Andescavage (2017b)	87, 79*	18 - 39	Volumes and growth of ventricles, extra-axial CSF, TBV, posterior fossa, brainstem parenchyma	Males faster growth trajectory of LVs. No difference in other volumes.
Conte (2018)	169^†^	20 - 24	Linear measurements of FOD, BPD, CC, cerebellum, brainstem	Males larger FOD and TCD. No significant difference in growth rate.
Cook (2023)	95^†^	19 - 40	FC (machine learning sex classification algorithm) across 200 ROIs	Fetal sex classified with 73% accuracy, primarily weighted by connections involving amygdala, caudate, frontal gyri, and somatomotor regions. Males greater within-region connections; females greater cross-region connections.
Correa (2023)	12, 17, 1^†^	31 - 37	Volumes of amygdala, putamen, caudate, hippocampus, cerebellum, thalamus; FC	No significant sex difference in volumes or FC.
Cortes-Albornoz (2023)	18, 13	18 - 41	Volumes and growth of intracranial and supratentorial parenchyma, brainstem cerebellum, intraventricular CSF, extra-axial CSF	No significant sex difference in volumes or growth trajectories.
De Asis-Cruz (2020)	13, 12	26 - 34	FC (seed-based connectivity strength across 46 ROIs)	No significant sex differences.
De Asis-Cruz (2021)	46, 49	19 - 40	FC (modularity, local efficiency, normalized clustering coefficient, small world index)	No significant sex differences.
Gafner (2023)	300, 298	28 - 37	Linear measurements of CC, FOD, BPD, TCD	Males larger FOD and BPD. No difference in CC or TCD.
Griffiths (2023)	109, 91	18 - 37	ICV; volumes of ventricles, brain parenchyma, extra-axial CSF; hemispheric asymmetry	Males greater volumes and growth trajectories in ICV and brain parenchyma. No difference in asymmetry.
Jacob (2011)	20^†^	21 - 32	Total hippocampal volume	No significant sex difference.
Jaimes (2020)	19, 13	28 - 37	DTI measures of commissural, association and projection tracts (volume, FA, and ADC)	No significant sex differences.
Ji (2023)	67, 53	25 - 39	FC coactivation patterns with supplementary motor area	No significant sex differences.
Kasprian (2011)	166^†^	18 - 37	Temporal lobe length, depth of superior temporal sulcus, and asymmetry of these structures.	No significant sex differences.
Kyriakopoulou (2017)	57, 51	21 - 39	Volumes: supratentorial brain tissue, LVs, cortex, cerebellum, extra-cerebral CSF. Linear: BPD, FOD, head circumference, TCD, atrial diameter, and vermis height, width, area.	Males larger total ventricular volumes. No significant differences in linear measures except TCD.
Liu (2024)	26, 29	19 - 40	Graph theory analysis of FC matrix properties based on 90 ROIs.	Males higher clustering coefficient and local efficiency. No differences in small-world properties, global efficiency, or nodal parameters.
Lu (2021)	75, 69	24 - 39	Volumes of total brain, CGM, WM, DGM, cerebellum, brainstem; Lobar surface areas, GI and sulcal depth	Males greater TBV, CGM, and brainstem volumes. No difference in cerebellar volume, local GI, or sulcal depth.
Lu (2022)	100, 91^‡^	26 - 35	Volumes of total brain, CGM, WM, DGM, cerebellum, brainstem, hippocampus; Lobar surface areas, GI and sulcal depth	No sex differences after accounting for multiple comparisons
Machado-Rivas (2022)	40, 58	20 - 38	Volumes and growth of 18 bilateral cortical, subcortical and developmental structures	Male larger ICV, LVs, brain parenchyma, DGM, CC, thalamus, and cerebellar hemispheres, plus growth rate of caudate.
Ren (2022)	91, 97	19 - 37	Volumes: TBV, ICV, CGM, subcortical brain, LVs, cerebellum, brainstem, extra-cerebral CSF	Males larger LVs. Other volumes not significantly different.
Righini (2006)	35, 27	20 - 37	Hippocampal infolding angle	No significant sex differences.
Schwartz (2021)	10, 7	20 - 35	Cortical thickness, surface area, local GI across 9 cerebral areas; hemispheric asymmetry	No significant sex differences.
Scott (2011)	19, 20	21 - 31	Supratentorial volume; volumes and growth of cortical plate, subplate and intermediate zone, germinal matrix, deep gray nuclei, ventricles	No significant sex differences.
Shi (2020)	357, 280	22 - 40	BPD, FOD, head circumference, TCD, cerebellar area, LCC, CC area, vermis height and area	Males larger BPD, FOD, and head circumference from 31–35 weeks.
Studholme (2020)	81, 81	18 - 37	Volumes and growth of TBV, ICV, ventricles, sulcal CSF, CGM, WM, DGM, cerebellum; lobular measures: GM, WM, surface area, curvature; asymmetry	Males greater WM growth and raw volumes for all measures except sulcal CSF. Females greater lobe curvature and local volumes (splenium, insula, occipital cortex) after controlling for ICV.
Thomason (2018)	59, 37	33.1 ± 4.1^§^	FC of sensorimotor network	No sex differences.
Tilea (2009)	206, 166	26 - 40	Bone and cerebral BPD; FOD; LCC; TCD; cerebellar height; vermis width and surface area	Males larger BPD. Females longer LCC after 31 weeks.
van den Heuvel (2023)	55, 34	21 - 40	Positive and negative FC between amygdala and 5 major anatomical clusters	No significant sex differences.
Vasung (2020)	27, 15	16 - 37	Regional volumes of 22 fetal cortical and transient fetal brain compartments	Males greater relative volumes of the left IFG, subplate of IFG, cingulate gyrus, and calcarine cortex. All other regions and all growth rates did not differ.
Vossough (2013)	27, 21	25 - 35	Segmented brain volumes	No significant sex differences.
Wheelock (2019)	70, 48	26 - 40	Correlation between GA and clustering across 16 FC networks, using graph theory and community detection analysis.	Stronger association between GA and intracerebellar FCs in males; stronger association between GA and posterior-cingulate-temporal pole and frontal-cerebellar FCs in females.
Wu (2020)	67, 52	24 - 40	Volumes and growth of TBV, CGM, DGM, WM, cerebellum, brainstem, hippocampus	Males greater volumes of TBV, CGM, DGM, WM, brainstem; faster growth of TBV and CGM.
	56, 43		Cortical folding (local GI, sulcal depth, curvedness)	No significant sex difference in cortical folding.
Ye (2020)	43, 49	21 - 38	Cerebellar vermis supero-inferior and antero-posterior diameters and area; cerebellar width and volume; superior cerebellar cistern width; cerebello-medullary cistern width	Males greater cerebello-medullary cistern width. Females larger vermis antero-posterior diameter. No significant difference in other posterior fossa measures.
Yun (2022)	61, 43	19 - 36	Timing and variability of sulcal emergence	No significant sex differences in sulcal emergence or variability after FDR correction.

All MRI studies (listed alphabetically by first author and year) reporting on sex differences in the fetal brain in utero. Gestational age (GA) of scanning is reported in range of weeks unless otherwise indicated.

* Same sample as Andescavage et al. (2017a) but reported different outcome measures.

† Number males (M) and females (F) not reported.

‡ Apparent expansion of 2021 paper by same authors.

§ Mean ± SD (range not reported).

ADC (apparent diffusion coefficient); BPD (biparietal diameter); CC (corpus callosum); CGM (cortical gray matter); CSF (cerebro-spinal fluid); DGM (deep gray matter); DTI (diffusion tensor imaging); FA (functional anisotropy); FC (functional connectivity); FDR (false discovery rate); FOD (fronto-occipital diameter); GI (gyrification index); GM (gray matter); ICV (intracranial volume); IFG (inferior frontal gyrus); LCC (length of CC); LV (lateral ventricles); ROI (region of interest); TBV (total brain volume); TCD (transverse cerebellar diameter); WM (white matter).

**Table 2.** Meta-analytic findings without and with imputed data.

A. Linear measures	Pooled effect size (quant. only)	Pooled effect size (all studies)
Cerebral FOD	**g = 0.249, p < 0.001** (κ = 3)	**g = 0.180, p = 0.007** (κ = 5)
Cerebral BPD	**g = 0.218, p < 0.001** (κ = 3)	**g = 0.156, p = 0.005** (κ = 5)
CC length	**g = 0.142, p = 0.025** (κ = 4)	**g = 0.142, p = 0.025** (κ = 4)
Cerebellar diameter	g = 0.129, p = 0.207 (κ = 5)	g = 0.129, p = 0.207 (κ = 5)
Vermis height	g = -0.033, p = 0.816 (κ = 3)	g = -0.026, p = 0.722 (κ = 5)
B. Volumes	Pooled effect size (quant. only)	Pooled effect size (all studies)
Lateral ventricles	**g = 0.452, p < 0.001** (κ = 4)	**g = 0.397, p < 0.001** (κ = 6)
ICV	**g = 0.379, p = 0.004** (κ = 6)	**g = 0.343, p = 0.004** (κ = 7)
Deep GM	**g = 0.352, p = 0.024** (κ = 3)	g = 0.178, p = 0.080 (κ = 6)
TBV	**g = 0.348, p = 0.008** (κ = 4)	**g = 0.269, p = 0.029** (κ = 5)
Brainstem	**g = 0.301, p = 0.015** (κ = 3)	g = 0.154, p = 0.074 (κ = 6)
Cortical GM	**g = 0.275, p = 0.012** (κ = 3)	**g = 0.207, p = 0.043** (κ = 4)
Hippocampus	**g = 0.260, p = 0.047** (κ = 3)	g = 0.143, p = 0.140 (κ = 4)
Cerebellum	g = 0.186, p = 0.137 (κ = 4)	g = 0.101, p = 0.178 (κ = 7)

Studies for which effect sizes were imputed (see Section 2.2) were added to studies reporting full quantitative (“Quant. only”) data (Figs. 2 and 3) in conducting meta-analyses of fetal linear and volumetric regional brain sex differences. The additional studies in the “All studies” column include: Tilea et al. (2009), Scott et al. (2011), Kyriakopoulou et al. (2017), and Cortes-Albornoz et al. (2023). Positive g values are larger in males. Bolding indicates p < 0.050.

**Table 3.**
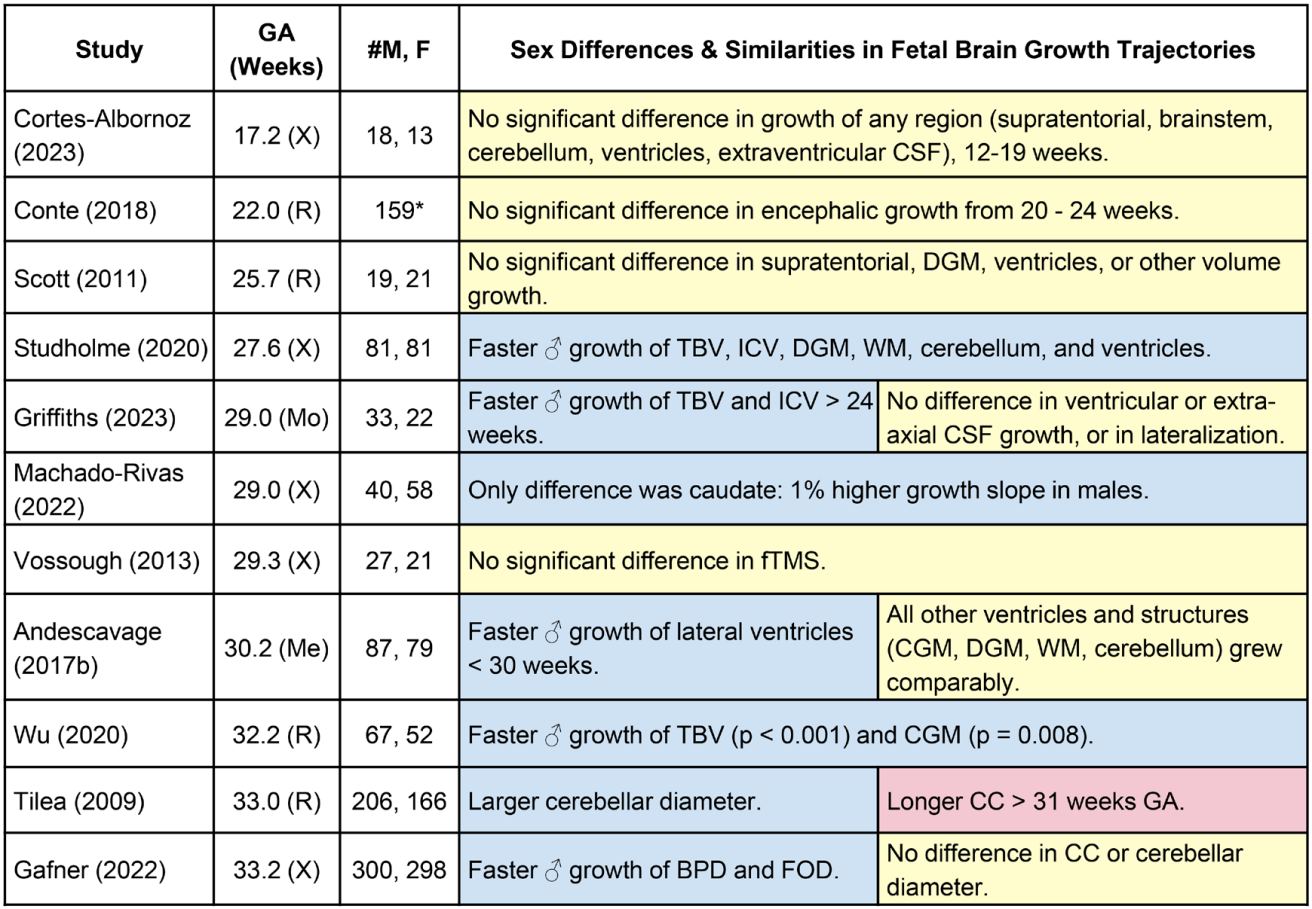
Sex differences and similarities in fetal brain growth trajectories.

Studies reporting on sex difference in fetal brain growth trajectories, ordered by gestational age (GA). Me = median GA; Mo = modal GA; R = midpoint of GA range; X = mean GA.

* Number males and females not reported.

BPD (biparietal diameter); CC (corpus callosum); CSF (cerebrospinal fluid); CGM (cortical gray matter); DGM (deep gray matter); fTMS (fetal total maturation score); FOD (fronto-occipital diameter); ICV (intracranial volume); TBV (total brain volume); WM (white matter).

Cell shading: blue = M>F; pink = F>M; yellow = no significant sex difference.

**Table 4.**
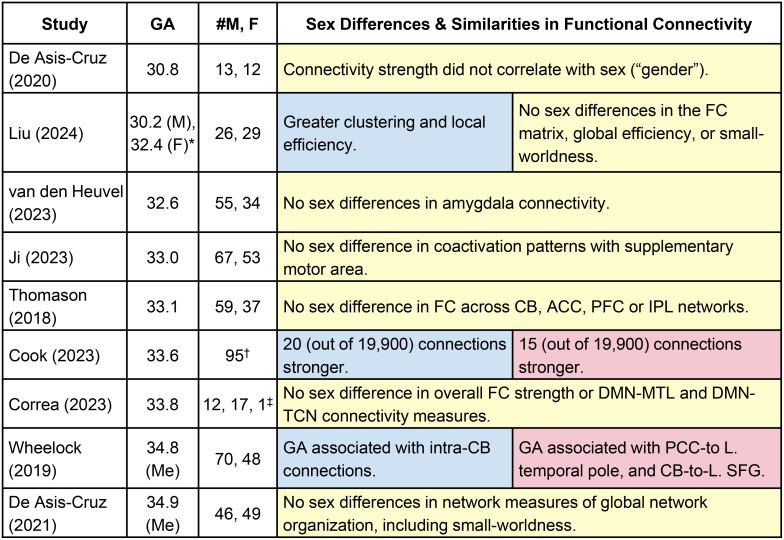
Functional connectivity comparison between male and female fetuses.

Studies reporting on sex difference in fetal brain functional connectivity (FC), ordered by mean gestation age (GA) in weeks, unless otherwise reported as median GA (Me).

* Significantly different, p = 0.011.

† Number males (M), females (F) not reported.

‡ Sex of one fetus unknown.

ACC (anterior cingulate cortex); CB (cerebellum); DMN (default mode network); IPL (inferior parietal lobule); MTL (medial temporal lobe); PCC (posterior cingulate cortex); PFC (prefrontal cortex); SFG (superior frontal gyrus); TCN (thalamocortical network).

Cell shading: blue = M>F; pink = F>M; yellow = no significant sex difference.

Outside of these four clusters, our search captured several other reports of prenatal sex comparisons which we did not analyze further due to insufficient replication. These measures include: diffusion tensor imaging of white matter tracts (Jaimes et al., 2020); temporal lobe length, sulcal depth, and asymmetry (Kasprian et al., 2011); hippocampal infolding angle (Righini et al., 2006); cortical thickness, surface area, gyrification, and hemispheric asymmetry (Schwartz et al., 2021); transient fetal brain compartments, cingulate and calcarine cortical volumes (Vasung et al., 2020); cortical folding (Wu et al., 2020); and timing and variability of sulcal emergence (Yun et al., 2022). In all of these studies except Vasung et al. (2020), no significant differences were found between male and female fetal brains (Table 1).

### 3.1 Linear brain measures

Turning to analyses for which there were sufficient replications, six studies reported data on sex differences in two-dimensional, or linear brain measurements, with the number of studies ranging from κ = 3 to 5 (for quantitative reports) and 4 to 5 when qualitative findings were included. These measures include: cerebral fronto-occipital diameter, cerebral biparietal diameter, corpus callosum length, cerebellar transverse diameter, and vermis height. Figure 2 shows forest plots for meta-analyses of each of these measures, with the findings sequenced from largest (fronto-occipital diameter; Hedges’s g = 0.249, p < 0.001) to smallest (vermis height; g = -0.033, p = 0.816) pooled effect sizes. All of the measures were larger in males except for vermis height. However, the effect sizes are all small, by Cohen’s criterion, and not statistically significant for either cerebellar measure (diameter or vermis height). Pooled effect sizes were smaller still, ranging from g = 0.180 to g = -0.026 when we included studies with only qualitative findings—that is, for which a null effect size was imputed based on the report of non-significant findings and insufficient data to calculate a precise effect (Table 2A).

### 3.2 Volumetric brain measures

Among the MRI studies comparing fetal male and female brain volumes, 10 studies (13 independent samples) reported global or regional prenatal brain volumes, with the number ranging from κ = 3 to 6 (for quantitative reports) and up to 7 when imputed findings were included. In this case, all measures were larger in males, with effect sizes ranging from Hedges’s g = 0.452 (p < 0.001) for lateral ventricular volume to g = 0.186 (p = 0.137) for cerebellar volume. Compared to linear measures, sex difference effect sizes were generally larger for volumetric measures and were statistically significant for all structures except the cerebellum (Fig. 3). Pooled effect sizes were once again smaller, ranging from g = 0.397 to g = 0.101 when we included studies reporting only qualitative findings (for which a null effect size was imputed based on a statement of “no significant sex difference”). In this case, sex differences in fetal deep gray matter, cortical gray matter, and hippocampal volume were not statistically significant (Table 2B).

In addition to these findings, four studies captured by our search reported on sex differences in white matter volumes. However, only two of these provided quantitative data so this data was not subjected to meta-analyses. Of these four studies, two reported significantly larger white matter volume in males, whereas two (Lu et al., 2021; 2022) found no significant sex difference. Among the positive findings, Wu et al. (2020) reported a 5% greater white matter volume in male fetuses, which was statistically significant, albeit without controlling for multiple comparisons. Studholme et al. (2020) found 7.4% greater white matter volume in male fetuses that remained significant after correcting for multiple comparisons; this was comparable to the 6.2% greater total brain volume (TBV) and 5.5% greater intracranial volume (ICV) in the same study. Of note, growth plots in Studholme et al. (2020) show that male and female curves for white matter volume diverge after about 30 weeks GA.

### 3.3 Brain growth trajectories

The fact that both linear and volumetric brain measures were greater in males, compared to females of the same gestational age, indicates that growth must be more accelerated in male fetuses. Several studies addressed this directly, focusing on rate of growth in addition to static linear or volume measures. Due to the heterogeneity of such reports, it was not possible to meta-analyze these findings, but all collected results are summarized in Table 3. Out of 11 studies that reported on fetal brain growth trajectories, six found at least some evidence for significantly faster brain growth in males, four found no significant sex differences in growth rate (Andescavage et al., 2017a; Conte et al., 2018; Cortes-Albornoz et al., 2023; Vossough et al., 2013), and one (Tilea et al., 2009) reported mixed results, with slightly faster growth of cerebellar diameter in males and faster growth of corpus callosum length in females, both of which were deemed “clinically insignificant.”

Of note, studies that failed to find a sex difference in the rate of brain growth generally included younger fetuses (<26 weeks GA) than those finding a significant sex difference, with minor exceptions. The most replicated finding (κ = 3) was faster growth of TBV in male fetuses after 27 weeks GA (Griffiths et al., 2023; Studholme et al., 2020; Wu et al., 2020). Other findings similarly corroborate static measures, including faster growth of ICV (Griffiths et al., 2023; Studholme et al., 2020) and lateral ventricles (Andescavage et al., 2017b; Studholme et al., 2020) in male fetuses, also after 27 weeks.

### 3.4 Functional connectivity

In addition to structural imaging, MRI has now been used to study the emergence of brain function before birth. Correlation between rs-fMRI and EEG measures of neuronal activity and neurovascular coupling in preterm infants supports the utility of fMRI during later gestation (Arichi et al., 2017), and mixed-sex studies have shown that the basic functional architecture of the brain emerges before birth, including primary motor, sensory, and nascent higher cortical networks (Turk et al., 2019). Given the emerging difference in brain size and regional volumes between male and female fetuses in the third trimester, fMRI studies offer an opportunity to test whether this structural difference is functionally significant.

Our systematic search captured nine studies that have reported on fetal sex differences using rs-fMRI during movement-free epochs. The average or median gestational age in these studies ranged from 31 to 35 weeks (all within the third trimester), and all used some version of functional connectivity (FC) analysis to map the pattern and properties of emerging cortical networks (Table 4). Despite the sex difference in brain size at this age, six of the nine studies found no difference in FC networks or properties, including connectivity strength (Correa et al., 2023; De Asis-Cruz et al., 2020), network organization (Ji et al., 2023; Thomason et al., 2018; van den Heuvel et al., 2023), global modularity and efficiency (De Asis-Cruz et al., 2021), and small-worldness (De Asis-Cruz et al., 2021; Liu et al., 2024). By contrast, three studies did report some type of sex difference in fetal FC, but all used different analytic methods and so cannot be considered replications. The most explicit claims about sex difference were by Cook et al. (2023) who identified 35 features (out of 19,900) that contributed significantly to the algorithmic discrimination of male and female fetal brains. These features were distributed across the brain, such that no particular brain region exhibited a greater pattern of hyper- or hypoconnectivity in males or females, nor was there a difference in the number of cross hemispheric connections between sexes. By contrast, Wheelock et al. (2019) used community detection analysis on rs-fMRI data and found different patterns of development (that is, GA-connectivity correlations) between sexes for 3 out of 136 FC nodes, with some hint of greater local connectivity in males versus greater cross-region connectivity in females. This finding roughly agrees with the graph theory analysis by Liu et al. (2024) who found greater local efficiency and clustering in males, but no difference in global efficiency or in the overall FC matrix. However, the males and females in Liu et al. (2024) were not appropriately matched for gestational age, limiting the relevance of these findings.

In sum, most evidence points away from any meaningful sex differences in functional connectivity before birth, although there are hints of a difference in local versus cross-region connectivity that could be explained by the sex difference in brain size (see Section 4.2).

### 3.5 Postmortem MRI studies

Finally, we report here on findings of five MRI studies performed on post-mortem fetuses. Although these studies met exclusion criteria because the fetuses were neither “healthy” nor “in utero,” they do provide insight into earlier gestation and have the advantage of imaging at higher resolution and without contamination by fetal movement. According to this postmortem imaging (Table 5), male and female brains do not differ with regard to gyrification, sulcal development, or subcortical structural volumes during the second trimester. Although some of the sample sizes in these studies are smaller than the in vivo studies discussed above, their technical advantages and focus on mid-gestation strengthen confidence in the conclusion that basic structural brain sex differences do not emerge before the third trimester of gestation. Of note, the lack of sex difference in cortical folding, or gyrification and sulcal development was replicated in studies of somewhat older live fetuses included in Table 1 (Lu et al., 2021, 2022; Schwartz et al., 2021; Wu et al., 2020; Yun et al., 2022).

**Table 5.** No sex difference in brain measures reported in post-mortem fetal MRI studies.

Study	GA	#M, F	Magnet strength	Brain measures	Significant sex differences?
Zhang (2010)	14–40	67, 64	3 T	Lengths of folded and unfolded cortical margins; degree of cortical folding	None
Meng (2012)	12–22	69*	7 T	Volumes of germinal matrix, caudate nucleus, lentiform nucleus, and dorsal thalamus	None
Zhang (2013)	12–22	31, 38	7 T	Presence of sulci on a 3D visualization model	None
Zhang (2016)	11–22	22, 23	7 T	Emergence of central sulcus on 3D visualization model	None
Yoshida (2017)	16–40	10, 12	1.5 T	Gyrification index of cortical folding	None

Results of five independent studies reporting on male-female brain structural differences using MRI of post-mortem fetuses.

* Number of M, F not specified.

## 4 Discussion

Quantitative MRI research on the human fetal brain took off about 20 years ago, following proof of its safety and improvements in imaging speed and motion correction (Studholme, 2011). Such advances have allowed researchers to better characterize normal prenatal development and clinicians to more accurately detect brain anomalies. As part of this normative data collection, many studies have included comparisons of male and female fetuses, but the present analysis is the first, to our knowledge, to systematically review MRI findings on sex differences in fetal brain structures and function.

### 4.1 The fetal brain is larger in males than females

Our search identified 34 studies with published brain measures comparing male and female fetuses matched for gestational age. Of these, 28 studies included measures that were replicated across at least three independent studies and so were included in meta-analyses and data summaries. Results provide clear evidence for larger size and faster brain growth in male compared to female human fetuses. The effect sizes of sex differences were larger for brain volumes than for linear measures (Table 2) and also larger for bigger structures (e.g., TBV) compared to smaller (e.g., brainstem) regions. Pooled effect sizes were smaller and fewer were significant when data were included from studies reporting null results without full quantitative data (Table 2, right column). Overall, we can conclude that the lateral ventricles, ICV, TBV, and cortical gray matter volumes are larger in male, compared to female fetuses, and the same is true for linear measures of fronto-occipital diameter, biparietal diameter, and corpus callosum length.

With regard to gestational age, data on linear brain measures (Fig. 2) show few trends between 22–33 weeks GA. By contrast, the effect sizes for sex difference in brain volumetric measures generally increase between 20 and 33 weeks (Fig. 3), with most of the significant differences coming in studies of third-trimester fetuses, as compared to those 27 weeks or younger GA. These findings also align with Scott et al. (2011), who reported “no sex difference” in supratentorial brain volume in the second trimester (p = 0.999). While that study met inclusion criteria, it could not be added to our meta-analysis because supratentorial volume is a truncated measure of TBV. (It excludes the cerebellum and brainstem.)

Results from our volumetric meta-analyses agree with overall findings from the 11 studies reporting on growth trajectories (Table 3), which revealed no significant sex differences before a mean/median age of 27.6 weeks but mostly reliable findings of faster male brain growth across the third trimester. These growth trajectory studies found faster male growth in the lateral ventricles and in global brain measures (TBV, ICV, biparietal diameter, and fronto-occipital diameter), with less reliable growth differences between males and females in smaller structures (cerebellum, deep gray matter, white matter, and the corpus callosum).

These MRI findings largely agree with prenatal ultrasound scanning which, based on its more widespread clinical use, has produced very large normative datasets. Thus, Melamed et al. (2013) found about a 2% greater biparietal diameter in male fetuses between 20–30 weeks GA, along with 1.6% greater male fronto-occipital diameter and head circumference in a large ultrasound database. Broere-Brown et al. (2016) also documented greater head circumference in male fetuses from the second trimester onward, and Galjaard et al. (2019) found significant differences in head circumference and biparietal diameter based on ultrasound measures from 15 weeks onward, amounting to about a 3-day growth advantage for male fetuses. These ultrasound studies range in size from 8,556 to 12,132 fetuses, so have greater power to detect sex differences in the much smaller brains of second-trimester fetuses.

Ultrasound has also been used to estimate brain volumes. Thus, Namburete et al. (2023) produced an atlas of brain growth based on 3D ultrasound that affirms 6% larger TBV in males by 22 weeks GA. However, that study found no significant sex differences in specific brain structures (e.g., cortical plate, cerebellum) after controlling for this global size. In other words, all brain structures appear to be proportionally larger in males from at least mid-gestation onward. A somewhat smaller sex difference in ICV was reported by de Zwarte et al. (2024) using automated ultrasound measures; the difference amounted to about a 1% larger ICV in males at 20 weeks, increasing to about 5% larger at 30 weeks GA.

Taken together with the present MRI findings, existing data are largely consistent in finding larger heads and intracranial contents in male fetuses, including ventricles, whole brain, and regional brain structures. The differences are apparent by the second trimester based on large ultrasound studies, but not apparent until the third trimester based on the smaller data collections permitted by MRI. Male brains appear larger than female brains across all structures, although the effect sizes are generally smaller for smaller structures. High-resolution post-mortem MRI studies (Table 5) similarly indicate no sex differences in cortical folding, gyrification and sulcal development, in agreement with the five in vivo studies that reported on these architectural features (Lu et al., 2021, 2022; Schwartz et al., 2021; Wu et al., 2020; Yun et al., 2022; Table 1).

### 4.2 No sex difference in fetal brain functional connectivity

In contrast to the brain structural size difference, MRI studies of fetal brain activity provide little evidence for prenatal sex difference. Recent research has demonstrated that functional brain networks do emerge before birth, beginning with primary sensory networks and progressing to higher-order circuits, including the default mode and thalamocortical networks by the third trimester (Correa et al., 2023). However, none of the nine studies captured in our systematic review found sex differences in the overall patterning of the canonical FC resting networks, nor did six of the nine find sex differences in other FC metrics. The three remaining studies reported mostly minor and varying findings with regard to sex: Cook et al. (2023) set up an algorithmic discrimination of male from female fetal brains that performed at 73% accuracy (versus 50% chance) based on a small handful of connections spread across the brain. However, those connections differed from those previously identified by Wheelock et al. (2019), who found just 3 out of 136 connections to differ significantly by sex, and then only in their association with gestational age, not in strength or network pattern. The third study, by Liu et al. (2024), reported overall network similarity between male and female fetuses, except for somewhat higher efficiency for local processing in select areas by males, relative to females. Unfortunately, the sexes were not adequately matched for gestational age in Liu et al. (2024), limiting interpretability of this finding.

Importantly, none of the studies assessing FC sex differences controlled for brain size, which as we’ve seen, differs as early as the second trimester. Resting brain activity patterns are highly correlated with total intracranial volume (Qing & Gong, 2016), indicating that larger brains exhibit different patterns of functional connectivity than smaller brains. Indeed, the algorithmic discrimination of male and female brains based on FC pattern is highly sensitive to brain size, such that sex prediction accuracy declines substantially when brain size is controlled for (Zhang et al., 2018; but see Serio et al., 2024). Similarly, the relative efficiency of local versus cross-regional connections—the sex difference reported by Liu et al. (2024) and inferred by Wheelock et al. (2019)—is likely also affected by brain size, which favors higher local network clustering as brain size increases (Ardesch et al., 2022).

Overall, current research has not found reliable evidence of sex differences in functional brain connectivity between male and female fetuses, a finding largely in agreement with neonatal studies. Future research on this question should control for total brain size.

### 4.3 Comparison to neonatal studies

MRI studies of the neonatal brain predate fetal studies and have recently incorporated much larger sample sizes. Given the overall continuity of brain development from pre- to post-natal life (de Graaf-Peters and Hadders-Algra, 2006; Eyre et al., 2021), it is worth comparing the present prenatal findings with recent large-scale neonatal studies of brain sex difference.

With regard to structural differences, several studies agree in finding 6–8% larger ICV and TBV in males during the first month of life (Dean et al., 2018a; Geng et al., 2024; Khan et al., 2024; Knickmeyer et al., 2014, 2017). However, they disagree about whether any sex differences in regional brain volumes persist after controlling for total brain or head size. Thus, Dean et al. (2018a) reported greater volumes of every brain region in males at 1 month of age, but none of the differences survived correction after controlling for males’ 8% larger TBV (Dean et al., 2018b). The same was found in a recent sample of Chinese infants between birth and 3 months of age (Geng et al., 2024). Similarly, Knickmeyer et al. (2014) found no significant sex differences in regional brain volumes after controlling for the 6% difference in ICV; however, in a later study, the same group reported larger volumes in the temporo-parietal junction in females and the cingulum in males (Knickmeyer et al., 2017). More recently, Khan et al. (2024) showed that all regional sex differences are dramatically reduced when controlling for the 6% larger TBV, but 8 out of 48 regions survived this correction, specifically a 3% larger parahippocampal gyrus and 1% larger parietal lobe volume in females. Studies utilizing diffusion tensor imaging have similarly found isolated or conflicting sex differences in neonatal white matter tracts (Geng et al., 2012; Kumpulainen et al., 2023; Liu et al., 2011; Qiu et al., 2013; Saadani-Makki et al., 2019) with no reliable male-female differences before 2 years of age (Geng et al., 2012).

Turning to functional sex differences, several studies have now explored resting brain activity patterns and functional connectivity in neonatal brains and generally find that males and females are “remarkably similar” (Gilmore et al., 2018). Thus, Gao et al. (2015) found no sex differences across nine canonical FC networks between birth and age 2, except for a faster increase in connectivity between frontoparietal networks in boys. Similarly, Chen et al. (2021) found no sex differences in voxel-level connectivity and reported that “global and local patterns [of FC] were highly consistent across males and females” between birth and age 6, except for some alternating pacing in FC development by girls and boys up to age 2. Other studies have reported scattered FC sex differences in neonates, but they are constrained to a small minority of voxels and do not replicate later in infancy (Fenske et al., 2023) or across other studies of neonates (Eyre et al., 2021).

Overall, the present findings on the fetal brain agree with the larger and more technically-accessible studies of neonates: male fetuses and neonates have larger brains than females, but this difference holds across the brain, with the possible exception of the fetal cerebellum. Functional connectivity, by contrast, does not differ reliably by sex at pre- and perinatal ages. Modest sex differences in specific structures and connections may emerge later in life (Eliot et al., 2021), alongside gender-differentiated behaviors that also become significant later in childhood and adolescence (Blakemore et al., 2013).

### 4.4 Global sex difference in fetal size

The collected data on fetal and neonatal brain sex differences do point to one reliable finding: males’ brains grow faster from early in gestation, achieving a significantly larger total size than females’ well before birth. This is not surprising, given the higher average birthweight of boys (about 114 g; CDC - National Center for Health Statistics, 2024) and greater size of male bodies and brains after puberty (Eliot et al., 2021). However, the physiological basis of this early growth difference is largely unknown, nor is it clear whether the faster growth of males contributes to their greater medical and developmental vulnerability in early life (DiPietro & Voegtline, 2017; Meakin et al., 2021).

The sex difference in prenatal growth is not unique to the brain and begins as early as the blastocyst stage: in vitro pre-implantation studies have identified that XY embryos have a greater total cell mass compared to XX embryos (Meakin et al., 2021). By 8–15 weeks of gestation, crown-rump length is significantly greater in males (Bukowski et al., 2007). In addition to greater head and brain size, male fetuses have larger abdominal circumference (Melamed et al., 2013) and greater mass of all internal organs except the adrenal glands (Ryan et al., 2018; Waszak & Cieślik, 2003). This early growth divergence, which begins before sexual differentiation of the gonads, implicates a genetic, as opposed to hormonal mechanism, a conclusion supported by findings from two clinical populations: 1) complete androgen insensitivity syndrome (CAIS), where XY fetuses who lack a functioning androgen receptor and appear phenotypically female have male-typical birthweights; and 2) XX fetuses with congenital adrenal hyperplasia (CAH) who are exposed to male-typical levels of testosterone from the first trimester onward but have female-typical birthweights (Miles et al., 2010). Similarly for brain size, XY women with CAIS have male-typical brain volumes (Savic et al., 2017) and XX women with CAH have female-typical brain volumes, despite other evidence of physical and psychological virilization (Beltz et al., 2021).

These findings indicate that the overall growth differential between male and female fetuses originates early in development through the Y or X chromosomes, likely acting through sex differences in placental function. According to Meakin et al. (2021), there are 58 genes whose placental transcription differs between male and female embryos, with some evidence pointing to enriched protein translation in male versus immune function in female trophoblast cells. Glucocorticoid signaling also differs between male and female placentae, possibly contributing to faster growth in male fetuses versus resilience (reserve placental capacity) in female fetuses (Clifton, 2010). By contrast, gene transcription in the fetal brain shows limited sex differences, including scant influence of sex hormones (Buonocore et al., 2025), again indicating that any sex differences in this organ are not truly brain-specific, but rather a product of the faster overall growth of male fetuses.

### 4.5 Limitations

This study has several limitations. Our systematic review captured 34 studies that statistically compared male and female fetuses on specific MRI measures of fetal brain structure or function. However, we excluded many more MRI studies that reported on fetal brain measures but failed to disaggregate the data by sex. This is a common problem in sex- and gender-related research but should improve as more researchers comply with journal and funding agency mandates requiring such sex-disaggregated reporting (Eliot et al., 2023).

Compared to studies of adult, adolescent, and even neonatal brain sex differences, fetal studies tend to have smaller sample sizes and lower statistical power to identify male-female differences. Prenatal MRI scanning is generally limited to fetuses with clinical concerns, so researchers cannot recruit the large participant pools that are increasingly the norm in healthy brain MRI databases. Of the scans obtained in a given study, many cannot be included due to excessive fetal motion. In addition, many of the studies included in our data tabulation and meta-analyses reported a mixture of cross-sectional and longitudinal data––that is, some of the fetuses were scanned more than once in the study (e.g., Cook et al., 2023; De Asis-Cruz et al., 2021; Kyriakopoulou et al., 2017; Lu et al., 2021, 2022). Between this lack of independence, variation in the reporting of group gestational ages (mean, median, mode, or range), and the limited GA range of the scanned fetuses, we were unable to use meta-regression to fully assess changes in sex differences across gestation.

Sex difference effect sizes (Hedges’s g values) were generally greater for larger structures (e.g., TBV versus hippocampal volume) but this does not mean that the proportional difference between males and females differs across structures. Given the spatial resolution of MRI, smaller measures are necessarily less precise, making it more difficult to discriminate significant group-level differences in small, compared to larger structures. Hence, the lack of significance for the various cerebellar sex differences may reflect the limit of measurement resolution, not a true negative. This seems likely given that large neonatal studies have found that raw volumes of all cortical regions and subcortical structures, including the cerebellum, are significantly greater in males (Dean et al., 2018a; Khan et al., 2024).

Finally, for measures of brain growth trajectories (Table 3) and functional connectivity (Table 4), we were unable to perform meta-analyses due to the wide heterogeneity of measures reported. Despite being limited to vote-counting, these systematic collections are largely consistent in finding faster growth of male fetal brains (Table 3) but minimal sex difference in fetal function connectivity networks or activity patterns (Table 4).

## 5 Conclusions

Systematic review and meta-analyses of 34 fetal MRI studies confirm a sex difference in global brain size by the third trimester of gestation. All brain regions and structural measures were larger in males, although the difference did not reach statistical significance for smaller structures such as the cerebellum, brainstem and hippocampus. The brain grows faster in male fetuses, in parallel with other bodily and organ measures, but there is little evidence that specific brain areas or structures are disproportionately larger in either sex. Similarly, functional MRI studies of human fetuses find overall sex similarity in the pattern and strength of connectivity across the canonical resting networks. In sum, group-level brain sex differences in the fetus do not readily explain perinatal male vulnerability or support claims of advanced female brain maturity. Brain sex differences likely grow more discernable later in development as gender-differentiating experiences grow increasingly common and salient.

## Data Availability

All data generated or analyzed during this study are included in this published article.
